# Effects of physical exercise on the cartilage of ovariectomized rats submitted to immobilization

**DOI:** 10.1590/S1679-45082015AO3418

**Published:** 2015

**Authors:** José Martim Marques Simas, Regina Inês Kunz, Rose Meire Costa Brancalhão, Lucinéia de Fátima Chasko Ribeiro, Gladson Ricardo Flor Bertolini

**Affiliations:** 1 Universidade Estadual do Oeste do Paraná, Cascavel, PR, Brazil.

**Keywords:** Osteoporosis, Immobilization, Exercise therapy, Physical therapy modalities, Rats, Wistar

## Abstract

**Objective:**

To analyze the effects of physical exercise on cartilage histomorphometry in osteoporosis-induced rats subjected to immobilization.

**Methods:**

We used 36 Wistar rats that were separated into six groups: G1, G2 and G3 submitted to pseudo-oophorectomy, and G4, G5 and G6 submitted to oophorectomy. After 60 days at rest, G2, G3, G5 and G6 had the right hind limbs immobilized for 15 days, followed by the same period in remobilization, being free in the box to G2 and G5, and climb ladder to G3 and G6. At the end of the experiment, the rats were euthanized, their tibias bilaterally removed and submitted to histological routine.

**Results:**

There was significant increase in thickness of the articular cartilage (F(5;29)=13.88; p<0.0001) and epiphyseal plate (F(5;29)=14.72; p<0.0001) as the number of chondrocytes (F(5;29)=5.11; p=0.0021) in ovariectomized rats, immobilized and submitted to exercise. In the morphological analysis, degeneration of articular cartilage with subchondral bone exposure, loss of cellular organization, discontinuity of tidemark, presence of cracks and flocculation in ovariectomized, immobilized and free remobilization rats were found. In ovariectomized and immobilized remobilization ladder rats, signs of repair of the cartilaginous structures in the presence of clones, pannus, subcortical blood vessel invasion in the calcified zone, increasing the amount of isogenous groups and thickness of the calcified zone were observed.

**Conclusion:**

Exercise climb ladder was effective in cartilaginous tissue recovery process damaged by immobilization, in model of osteoporosis by ovariectomy in rats.

## INTRODUCTION

Menopause leads to a slow and gradual decrease of estrogen and progesterone, which increases bone resorption and decreases calcium absorption by bone tissue. This results in more fragile bones and increased predisposition to the onset of diseases, such as osteoporosis^(1,2)^ and osteoarthritis.^(3)^ Decreasing levels of estrogen shorten the half-life of cartilage and increase erosion of the cartilage surface,^(4)^ and this animal model has been a useful tool in studies investigating cartilage damage after ovariectomy.^(5)^

Immobilization is frequently used in musculoskeletal injuries, and can cause serious damage to the musculoskeletal system, particularly to the articular cartilage, resulting in temporary or permanent disability of the subject and increasing health care costs.^(6,7)^ Different treatments were proposed for rehabilitation after immobilization. However, exercise is preferred for improving muscle strength, preventing fractures and promoting tissue repair in the muscle, bone and cartilage.^(7-11)^

The rationale for this study considers the increased life expectancy of the population, the understanding that menopause can lead to some diseases, such as osteoporosis and osteoarthritis (very relevant public health issues), the disabilities and deleterious effects of immobilization on the tissue structure, and the need to gather scientific evidence for therapies commonly used in clinical practice.

## OBJECTIVE

To assess the effects of exercise on cartilage histomorphometry in female rats with induced osteoporosis subjected to immobilization.

## METHODS

### Experimental groups

We used 36 female nulliparous adult (10±2 weeks) Wistar rats, with mean weight of 317.2±22.1g, kept in polypropylene cages, *ad libitum* water and feeding, 12 hour light/dark photocycle, at controlled room temperature (25±1°C). The study was conducted according to international ethical standards of animal experimentation and approved by the Animal Research Ethics Committee of Unioeste under number 4,112.

The rats were randomized into six groups:

– G1 (n=6): rats subjected to sham ovariectomy (pseudo-ovariectomy), followed by 60 days with no intervention.

– G2 (n=6): sham ovariectomy and 60 days with no intervention. Then, the right hind limb (RHL) was immobilized for 2 weeks. Later, the rats were put on free remobilization for the same period and only came into contact with a ladder in the last 10 days.

– G3 (n=6): sham ovariectomy and 60 days with no intervention. Then, the RHL was immobilized for 2 weeks, and the rats were subsequently subjected to ladder climb exercise for 10 days, with a 2-day interval after day 5. They performed 10 climbs on week one (five days) and 20 on week two (5 days), with a 1-minute interval between climbs.

– G4 (n=6): bilateral ovariectomy and 60 days with no intervention.

– G5 (n=6): bilateral ovariectomy and 60 days with no intervention. Later, immobilization and remobilization were performed similarly as for G2.

– G6 (n=6): bilateral ovariectomy and 60 days with no intervention. Then, immobilization and remobilization procedures were performed similarly as for G3.

### Ovariectomy and sham ovariectomy protocol

For ovariectomy, sham ovariectomy, immobilization and euthanasia, the rats were weighed and subjected to an anesthesia protocol consisting of intraperitoneal injection of xylazine hydrochloride (12mg/kg) and ketamine hydrochloride (95mg/kg).

During the ovariectomy, after anesthesia, clipping was performed as well as antisepsis with iodinated alcohol in the lower abdomen, and then a longitudinal incision was made with a no. 11 surgical blade. At the peritoneal cavity, the adipose tissue was retracted until the surgeons could identify the fallopian tubes and ovaries. Suturing of the uterine horns was performed with a single 4.0 catgut suture, allowing for bilateral resection of the ovaries.

At the end of the procedure, internal and external sutures were placed using a single 4.0 catgut reabsorbable thread and 4.0 nylon, respectively. The pseudo- ovariectomy followed all surgical steps of the ovariectomy, except for removal of the ovaries. Following the surgery, the rats spent 60 days with no intervention, moving freely in the cage.

### Immobilization protocol

Immobilization was carried out according to the model proposed by Booth and Kelso,^(12)^ modified for just one limb, as proposed by Matheus et al.,^(13)^ with adaptation of the material used for the static cast brace. Prior to immobilization, anesthesia was performed, and then the RHL was wrapped in a tubular mesh with cotton bandage, from hip to ankle. Then fast-dry plaster wrap was used to mold a 50g contention brace, with the RHL at full knee extension and full plantar flexion of the ankle for the rats in G2, G3, G5 and G6.

### Remobilization protocol

After the immobilization was completed, the rats in G3 and G6 were subjected to the ladder climb exercise with 10 repetitions in the first week (five days) and 20 repetitions in the second week (5 days), with 1-minute intervals between climbs and 2-day intervals between weeks. The rats in G2 and G5 were allowed free remobilization in the cage, coming into contact with a ladder at 10cm from the dark box, only once, in the same period of the exercise performed with G3 and G6. The equipment used for the exercise consisted of a ladder with 67 steps separated by 1.5cm, 20.5cm wide, 118cm high and with a tilt angle of 80° (approximately). In the upper platform, there was a dark chamber of 28,5cm (length) x 18.5cm (height) x 15cm (width), available for the rats to rest between sets of climbs, and to attract them and stimulate them to perform the exercise.

### Histomorphometric and histomorphological analyses

At the end of the experiment, the rats were weighed, anesthetized and euthanized with decapitation by guillotine. Then, the right and left tibias were removed and subjected to the laboratory routine, *i.e.* diaphonized and embedded in paraffin; a microtome was used to make 7μm cuts, and the slides were prepared with frontal plane cuts and hematoxylin and eosin (HE) stain. The slides were photomicrographed in an Olympus DP71^®^ microscope at 3 points for assessment of articular cartilage, namely P1, P2 and P3, which correspond respectively to the lateral, intermediate and medial tibia. To analyze the epiphyseal plate, a photomicrograph was taken of the intermediate point. We used 200x magnification for thickness measurements of the articular cartilage and epiphyseal plate, and also for the chondrocyte count. The images were analyzed with the software Image Pro-Plus 6.0^®^. For the histomorphological analysis, in the articular cartilage, we observed the structure and cellular organization, such as changes in the articular surface.

### Statistical analysis

The study data were evaluated by comparing the results obtained for the left hind limb (LHL - control) and the RHL (immobilized), between rats in the same group and among groups. For this end, we used the software BioEstat 5.0 with values presented as mean and standard deviation. The paired Student *t*-test was conducted for comparison between the right and left sides within the same group, and one-way analysis of variance (ANOVA) was used for comparison between experimental groups, for the right and left sides. The level of statistical significance was p ≤0.05.

## RESULTS

### Articular cartilage thickness

**Figure 1:**
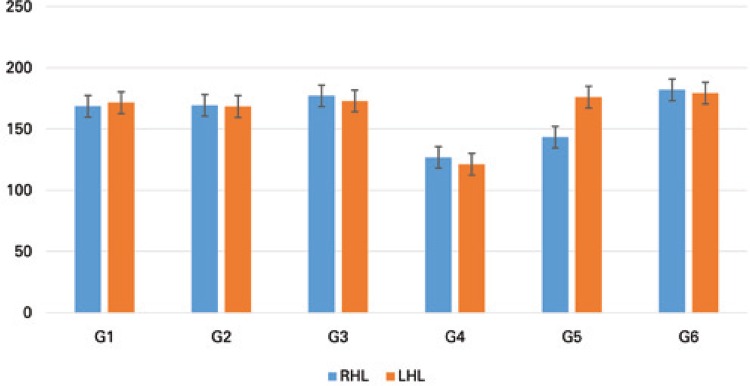
Thickness of the upper articular cartilage of the tibia, compared between rats in the different study groups (G1, G2, G3, G4, G5 and G6), and between the right (target) and left (control) hind limbs. The same letter represents similarities and different letters represent significant differences between experimental groups, for the same side

RHL: right hind limb; LHL: left hind limb.

For the articular cartilage thickness, there was a significant decrease in the RHL compared to the LHL in G5 (p=0.0138). In the intergroup comparison, significant reduction in the RHL was found for G4 when compared to G1, G2, G3 and G6, and for G5 when compared to G1 and G6 (F(5;29)=13.88; p<0.0001). The LHL showed significant decrease in G4 when compared to the other groups (F(5;29)=10.72; p<0.0001) (Figure 1).

### Epiphyseal plate thickness

**Figure 2:**
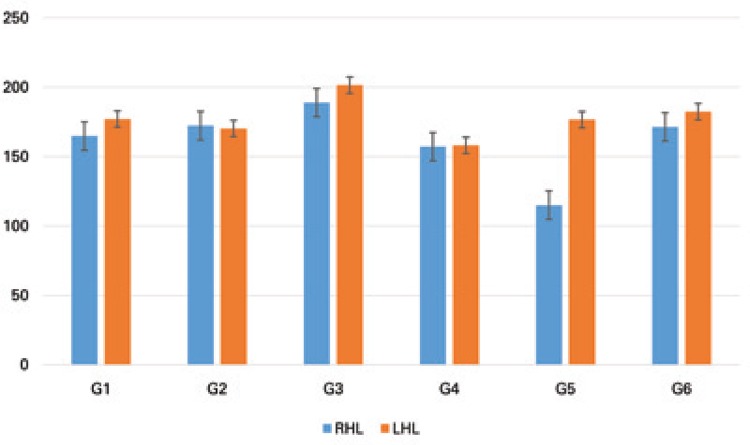
Thickness of the tibial epiphyseal plate e, compared between rats in the different study groups (G1, G2, G3, G4, G5 and G6), and between the right (target) and left (control) hind limbs. The same letter represents similarities and different letters represent significant differences between experimental groups, for the same side

RHL: right hind limb; LHL: left hind limb.

The analysis of the epiphyseal plate thickness showed a significant decrease in the RHL compared to the LHL in G5 (p = 0.0187). The intergroup comparison pointed to a significant reduction in G5 compared to the other groups, as well as higher values in G1 when compared to G4 (F(5;29)=14.72; p<0.0001) (Figure 2).

### Chondrocyte count

**Figure 3:**
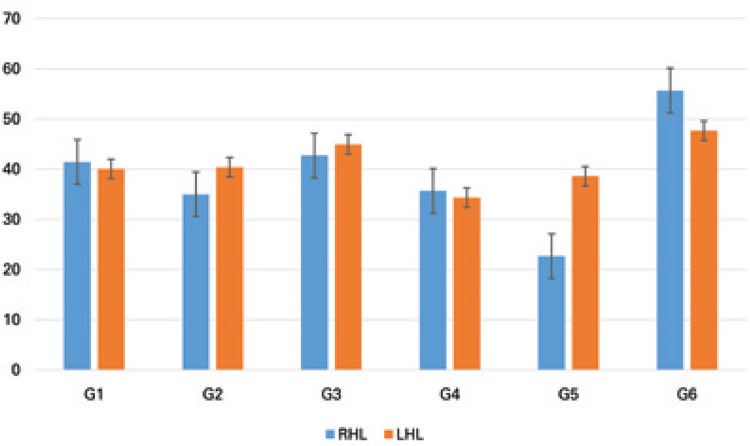
Chondrocyte count in the upper articular cartilage of the tibia (unit), compared between rats in the different study groups (G1, G2, G3, G4, G5 and G6), and between the right (target) and left (control) hind limbs. The same letter represents similarities and different letters represent significant differences between experimental groups, for the same side

RHL: right hind limb; LHL: left hind limb.

As for the number of chondrocytes, G5 was the only group with a significant decrease in the RHL when compared to the LHL (p=0.0006). The comparison between experimental groups showed a significant decrease in G2, G4 and G5 compared to G6, as well as G1 and G3 compared to G5, when comparing the right hind limbs (F(5;29)=10.16; p<0.0001). When comparing the left hind limbs, G6 had higher values compared to G1, G2, G4, and G5 (F (5; 29)=5.11, p<0.0021) (Figure 3).

### Histomorphology

**Figure 4:**
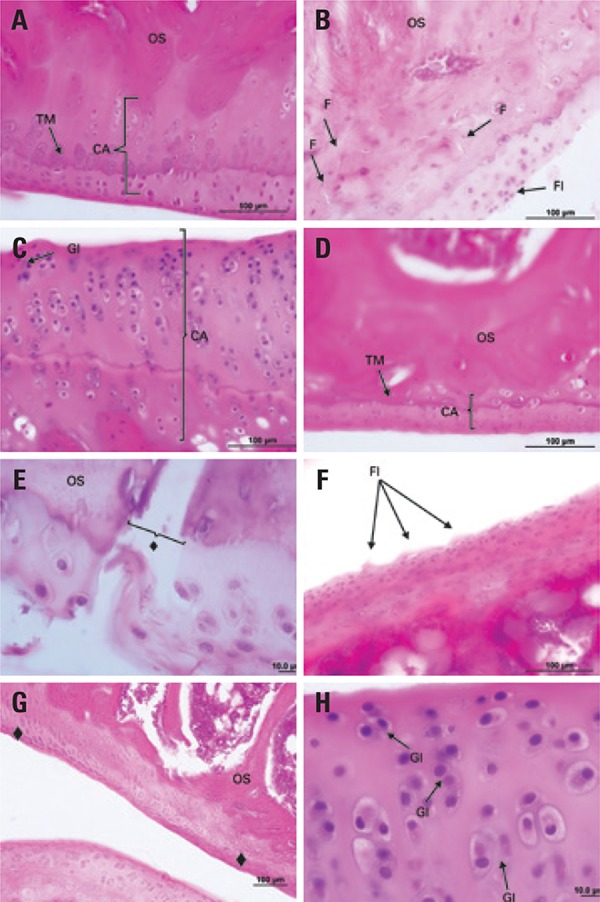
Photomicrographs of the upper articular cartilage from the right tibia of female rats, G1 (A), G2 (B), G3 (C), G4 (D), G5 (E and F) and G6 (G and H); frontal cut; hematoxylin and eosin stain. (A) Panoramic view of the articular cartilage (AC), with evidence of the tidemark (TM), and partial view of the subchondral bone (SB); (B) presence of cracks (C), flocculation (Fl) and tissue disorganization; (C) increased articular cartilage thickness, hypercellularity and increased number of isogenous groups (IG); (D) decreased articular cartilage thickness and normal cellular organization; (E) loss of articular cartilage (♦) with subchondral bone exposure; (F) presence of flocculation (Fl) on the cartilage surface; (G) restoration of the articular cartilage (♦) with pannus formation and presence of clones; (H) increased articular cartilage thickness, hypercellularity and increased isogenous groups

AC: articular cartilage; TM: tidemark; SB: subchondral bone; C: cracks; Fl: flocculation; IG: isogenous groups

In G1 (sham ovariectomy) and G4 (ovariectomy), no changes were observed in the thickness of the articular cartilage or the epiphyseal plate, and morphological aspects remained normal; however, in G4, there was significant reduction in the thickness and chondrocyte count, as shown in the histomorphometric analysis. In G2 (sham ovariectomy, immobilization and free remobilization), there were areas of articular cartilage degeneration, loss of cellular organization, flocculations, decreased chondrocyte count and some granulation tissue (pannus). In G3 (sham ovariectomy, immobilization and remobilization with ladder training), there were signs of repair of cartilaginous structures, with presence of cell clones and pannus. In G5 (ovariectomy, immobilization and free remobilization), there was evidence of degeneration of the articular cartilage, presence of cracks and flocculation, discontinuity of the tidemark, and exposure of subchondral bone. In G6 (ovariectomy, immobilization and remobilization with ladder training), there was pannus formation, subcortical blood vessel invasion into the calcified zone, as well as increase in the number of isogenous groups and the thickness of the calcified zone (Figure 4).

## DISCUSSION

The study results suggest that estrogen-deprivation induced by ovariectomy led to significant loss of articular cartilage. This hypoestrogenism changes the remodelation of cartilage tissue,^(4)^ favoring the onset of osteoarthrosis.^(1,5,6,14,15)^ This can be aggravated when immobilization is required, which may cause irreversible damage to the articular cartilage, *i.e.* the association of ovariectomy and immobilization may accelerate the onset of damage to the muscle and cartilage tissues.^(16-18)^

Immobilization leads to degeneration of the articular cartilage, with atrophic changes, decreased thickness, decreased synthesis of cartilage matrix proteoglycans, irregular cartilage surface, cartilage necrosis and ulceration, increased number of inflammatory cells, and lower total cartilage mass and volume.^(7-9,16,19-24)^ Some of these characteristics were observed in the group subjected to sham ovariectomy and immobilization, indicating that immobilization may have aggressive effects as early as at 2 weeks. Also, estrogen deficiency causes morphological changes in cartilage, leading to its degeneration,^(25)^ and this is supported by the observation of lower cartilage thickness in the left limb only in the ovariectomized group.

Exercise is indicated as a treatment for arthralgia resulting from post-menopausal osteoarthritis.^(26)^ This option has been the most advocated for remobilization since articular motion is capable of promoting physical, biochemical and histological changes that favor restoration of macromolecular synthesis and at least partial reversibility of cartilage damage.^(8,27,28)^ However, Portinho et al.^(29)^ used free remobilization for 15 days associated with stretching of the soleus muscle for 30 seconds three times daily, and did not see any changes in the articular cartilage thickness resulting from immobilization and remobilization. Del Carlo et al.^(9)^ found that immobilization for 45 days caused increase in the articular thickness and the number of chondrocytes, irregular articular surface and loss of cartilage matrix proteoglycans.

We did not find any studies investigating the cartilage-related effects on ovariectomized rats subjected to immobization with later remobilization with ladder climb training. However, some studies suggest this type of exercise can successfully promote improvement in bone mineral density and bone stiffness, and hypertrophy of the gastrocnemius, flexor digitorum longus, plantaris and triceps muscles.^(30-33)^

In the present study, the sham ovariectomized rats subjected to immobilization -either free remobilization (G2) or ladder training (G3) - were able to maintain the articular cartilage and growth plate thickness as well as the number of chondrocytes at levels comparable to those of the non-immobilized limb. This was also seen with the ovariectomized rats subjected to immobilization and remobilization with ladder training (G6). However, the same benefit was not seen in G5, which demonstrates that immobilization leads to significant losses of cartilage tissue in ovariectomized rats. Differently from the ladder training (G6), free remobilization was unable to reverse the deleterious effects on the cartilage tissue, probably because this would require weight-bearing on the joint. Remobilization with weight bearing on the joint promotes the secretion of proteoglycans in the matrix, allowing for restoration of the cartilage structure.^(8)^ Treadmill exercise was shown to be useful in ovariectomized rats to protect the articular cartilage.^(34)^

Damage to the extracelular matrix and complete degradation of the articular cartilage may result from the increased activity of matrix metalloproteinases.^(35)^ In the morphological analysis, we observed predominance of degraded areas, especially in G5 (ovariectomy, immobilization and free remobilization), and predominance of articular cartilage restoration in G2, G3 and G6, though more evident in the last two, due to the repairing effect of physical exercise. There was mainly the presence of pannus, increase in isogenic groups, and cell clones resulting from inflammation of joint tissues.^(36)^ These reactions reflect the restoration of the damaged articular cartilage, due to the proliferative capacity of chondrocytes and repair of the subchondral bone, with increased nutritional support in the region promoted by remobilization programs.^(9,36)^

## CONCLUSION

In short, ovariectomy associated with immobilization was observed to cause thinning of the articular cartilage and growth plate, decrease in the number of chondrocytes, articular cartilage degeneration, loss of cellular organization, flocculation and pannus. Free remobilization was not able to reverse the damage to the cartilage tissue of the tibia in ovariectomized and immobilized rats, but reversibility was achieved with ladder-based remobilization, evidenced by improvement of morphometric and morphological parameters with signs of repaired cartilaginous structures observed by the presence of cell clones and pannus, subcortical blood vessel invasion into the calcified zone, increased number of isogenous groups and increased thickness of the calcified zone.
