# Supplementary material for: Effects of physical exercise on the cartilage of ovariectomized rats submitted to immobilization
Source: Einstein (Sao Paulo). 2015 Oct-Dec;13(4):574–9. doi: 10.1590/S1679-45082015AO3418 (PMC4878633; doi:10.1590/S1679-45082015AO3418)
Supplement: Translation

# Translation: Efeitos do exercício físico sobre a cartilagem de ratas ooforectomizadas submetidas à imobilização

**Authors:** José Martim Marques Simas, Regina Inês Kunz, Rose Meire Costa Brancalhão, Lucinéia de Fátima Chasko Ribeiro, Gladson Ricardo Flor Bertolini

**Affiliations:** 1 Universidade Estadual do Oeste do Paraná, Cascavel, PR, Brasil.

**Keywords:** Osteoporose, Imobilização, Terapia por exercício, Modalidades de fisioterapia, Ratos Wistar

## Abstract

**Objetivo:**

Analisar os efeitos do exercício físico sobre a histomorfometria da cartilagem de ratas induzidas à osteoporose e submetidas à imobilização.

**Métodos:**

Foram utilizadas 36 ratas Wistar separadas em seis grupos: G1, G2 e G3 submetidas à pseudo-ooforectomia e G4, G5 e G6, à ooforectomia. Após 60 dias em repouso, G2, G3, G5 e G6 tiveram o membro posterior direito imobilizado por 15 dias, seguido pelo mesmo período em remobilização, sendo livres na caixa para G2 e G5, e de subida em escada para G3 e G6. Ao final do experimento, as ratas foram eutanasiadas, e suas tíbias foram retiradas bilateralmente e submetidas à rotina histológica.

**Resultados:**

Houve aumento significativo de espessura da cartilagem articular (F(5;29)=13,88; p<0,0001) e da placa epifisária (F(5;29)=14,72; p<0,0001), bem como do número de condrócitos (F(5;29)=5,11; p<0,0021) em ratas ooforectomizadas, imobilizadas e submetidas ao exercício em escada. Nas análises morfológicas, verificaram-se degeneração da cartilagem articular com exposição de osso subcondral, perda da organização celular, descontinuidade da *tidemark*, presença de fissuras e floculações em ratas ooforectomizadas, imobilizadas e com remobilização livre. Nas ratas ooforectomizadas, imobilizadas e com remobilização em escada, observaram-se sinais de reparação das estruturas cartilaginosas com presença de clones, *pannus*, invasão de vasos sanguíneos subcorticais na zona calcificada, aumento da quantidade de grupos isógenos e espessura da zona calcificada.

**Conclusão:**

O exercício físico de subida em escada mostrou-se efetivo no processo de recuperação do tecido cartilaginoso danificado pela imobilização, em modelo de osteoporose por ooforectomia em ratas.

## INTRODUÇÃO

A menopausa provoca a diminuição lenta e gradativa dos hormônios estrógeno e progesterona, produzindo aumento da reabsorção óssea e diminuição da absorção de cálcio pelo tecido ósseo. Dessa forma, os ossos ficam mais fragilizados e aumenta a predisposição ao aparecimento de doenças, como a osteoporose^(1,2)^ e osteoartrite.^(3)^ A diminuição dos níveis de estrógeno acelera a meia-vida da cartilagem e aumenta a erosão da superfície da cartilagem,^(4)^ sendo que o modelo animal tem se apresentado como útil ferramenta para estudos relacionados à lesão da cartilagem pós-ovariectomia.^(5)^

A imobilização é amplamente utilizada em lesões musculoesqueléticas, e pode gerar graves prejuízos ao sistema musculoesquelético e, particularmente, à cartilagem articular, além de acarretar em incapacitação temporária ou permanente do indivíduo e em elevação dos custos de cuidados em saúde.^(6,7)^ Vários tratamentos têm sido propostos para períodos de reabilitação pós-imobilização. No entanto, o exercício físico tem sido o mais preconizado, por melhorar a força muscular, prevenir fraturas e promover reparações na estrutura tecidual muscular, óssea e cartilaginosa.^(7-11)^

Assim justifica-se este estudo tendo em vista a elevação da expectativa de vida da população, o entendimento de que a menopausa pode levar a doenças como a osteoporose e osteoartrite (que são grandes problemas de saúde pública), as incapacitações e os efeitos deletérios nas estruturas teciduais decorrentes de uma imobilização, além da necessidade de tornar evidência científica recursos terapêuticos amplamente utilizados na prática clínica.

## OBJETIVO

Analisar os efeitos do exercício físico sobre a histomorfometria da cartilagem de ratas induzidas à osteoporose e submetidas à imobilização.

## MÉTODOS

### Grupos experimentais

Foram utilizadas 36 ratas da linhagem Wistar, adultas (10±2 semanas), nulíparas, com peso médio de 317,2±22,1g, mantidas em gaiolas de polipropileno com livre acesso à água e à ração, fotoperíodo claro/escuro de 12 horas e temperatura ambiente controlada (25°±1°C). O estudo foi realizado conforme as normas éticas internacionais de experimentação animal, sendo aprovado pelo Comitê de Ética em Experimentação Animal da Unioeste, sob o parecer 4.112.

As ratas foram separadas aleatoriamente em seis grupos:

– G1 (n=6): as ratas foram submetidas à cirurgia simulada de ooforectomia (pseudo-ooforectomia) e permaneceram 60 dias sem intervenção.

– G2 (n=6): pseudo-ooforectomia e permaneceram 60 dias sem intervenção. Após, foi realizada imobilização do membro posterior direito (MPD) por 2 semanas. Posteriormente, ficaram em remobilização livre pelo mesmo período, sendo apenas colocadas em contato com uma escada nos últimos 10 dias.

– G3 (n=6): pseudo-ooforectomia e permaneceram 60 dias sem intervenção. Após, foi realizada imobilização do MPD por 2 semanas, sendo posteriormente submetidas ao exercício de subida em escada durante 10 dias, com intervalo de 2 dias após a quinta sessão. Foram realizadas 10 subidas na primeira semana (5 dias) e 20 na segunda semana (5 dias), com intervalo de 1 minuto entre as subidas.

– G4 (n=6): cirurgia de ooforectomia bilateral e permaneceram 60 dias sem intervenção.

– G5 (n=6): ooforectomia bilateral e permaneceram 60 dias sem intervenção. Após, foi realizado imobilização e remobilização semelhante ao G2.

– G6 (n=6): ooforectomia bilateral e permaneceram 60 dias sem intervenção. Após, foram realizados procedimentos de imobilização e de remobilização, semelhante ao G3.

### Protocolo de ooforectomia e pseudo-ooforectomia

Para a realização da ooforectomia, pseudo-ooforectomia, imobilização e eutanásia, as ratas foram pesadas e submetidas a um protocolo de anestesia, que consistiu em injeção intraperitoneal de cloridrato de xilazina (12mg/kg) e cloridrato de cetamina (95mg/kg).

Na realização da ooforectomia, após realizada a anestesia, foram realizadas a tricotomia e a antissepsia com álcool iodado na região de baixo ventre, sendo, em seguida, feita a incisão cirúrgica longitudinal, com lâmina de bisturi número 11. Após acessar a cavidade peritoneal, foi afastado o tecido adiposo, até que se conseguissem identificar as tubas uterinas e ovários. Em seguida, realizou-se sutura com fio *catgut* 4.0 simples na área dos cornos uterinos, promovendo a ressecção dos ovários bilateralmente.

Ao final do procedimento, foram realizadas suturas internas com fio reabsorvível *catgut* 4.0 simples e as externas, com fio de nylon 4.0. A pseudo-ooforectomia consistiu na realização de todas as etapas cirúrgicas semelhantes à ooforectomia, com exceção da remoção dos ovários. Subsequente à cirurgia, as ratas permaneceram por 60 dias sem qualquer intervenção, livres na gaiola.

### Protocolo de imobilização

Foi realizada imobilização conforme modelo proposto por Booth e Kelso,^(12)^ sendo readequado para somente um dos membros, como proposto por Matheus et al.,^(13)^com adaptação do material utilizado para confecção de órtese estática gessada. Previamente à imobilização, realizou-se o procedimento anestésico, sendo, em seguida, realizada a MPD, desde o quadril até o tornozelo, envolvido por uma malha tubular juntamente de ataduras de algodão. Em seguida, foi moldada a órtese contensora, de aproximadamente 50g, utilizando-se atadura gessada de secagem rápida, com o MPD em extensão completa de joelho e tornozelo em flexão plantar máxima, nas ratas de G2, G3, G5 e G6.

### Protocolo de remobilização

Após a retirada da imobilização, as ratas de G3 e G6 foram submetidas ao exercício de subida em escada, com 10 repetições na primeira semana (5 dias) e 20 repetições na segunda semana (5 dias), com intervalo de 1 minuto entre as subidas e de 2 dias entre as semanas. As ratas de G2 e G5 realizaram remobilização livre na gaiola, sendo colocadas em contato com uma escada a 10cm da caixa escura, uma vez apenas, no mesmo período em que era realizado o exercício com G3 e G6. O equipamento utilizado para a realização do exercício físico consistiu de uma escada com 67 degraus, com espaçamento de 1,5cm entre os degraus da grade, 20,5cm de largura, 118cm de altura e ângulo de inclinação de 80° (aproximadamente). Na região superior, havia uma câmara escura de 28,5cm de comprimento, 18,5cm de altura e 15cm de largura, que servia para que as ratas descansassem entre as séries de subidas, e para que se sentissem atraídas para um refúgio e estimuladas a realizarem o exercício.

### Análise histomorfométrica e histomorfológica

Ao final do experimento, as ratas foram pesadas, anestesiadas e eutanasiadas por meio de decapitação em guilhotina. Após, foram retiradas as tíbias direitas e esquerdas, submetidas à rotina de laboratório, sendo diafanizadas e incluídas em parafina, com a realização de cortes de 7µm em micrótomo e a confecção das lâminas com cortes em plano frontal, que foram coradas em hematoxilina e eosina (HE). As lâminas foram fotomicrografadas em microscópio *Olympus *DP71^®^ em três pontos para as análises da cartilagem articular, denominados P1, P2 e P3, que correspondem respectivamente às porções lateral, intermédia e medial da tíbia. Para a análise da placa epifisária, foi realizada fotomicrografia do ponto intermediário. Foi utilizado aumento de 200 vezes para a realização de mensurações da espessura da cartilagem articular e placa epifisária, e também para a contagem do número de condrócitos. As imagens foram submetidas à análise por meio do programa Image Pro-Plus 6.0^®^. Para a análise histomorfológica, foram observadas, na cartilagem articular, a estrutura e a organização celular, como presenças de alterações na superfície articular.

### Análise estatística

Os dados da pesquisa foram avaliados por meio da comparação dos resultados obtidos no membro posterior esquerdo (MPE − controle) e MPD (imobilizado), entre as ratas do mesmo grupo e entre os grupos experimentais. Para isso, foi utilizado o programa BioEstat 5.0, com os valores apresentados em média e desvio padrão. Foi realizado o teste *t* de Student pareado para a comparação entre o lado direito e esquerdo, dentro do mesmo grupo, e Análise de Variância (ANOVA) unidirecional para comparação entre grupos experimentais para os lados direito e esquerdo. O nível de significância estatística considerado foi de p≤0,05.

## RESULTADOS

### Espessura da cartilagem articular

Para a espessura da cartilagem articular, observou-se diminuição significativa do MPD em relação ao MPE nas ratas do G5 (p=0,0138). Na comparação intergrupos, para o MPD, houve redução significativa do G4 em relação ao G1, G2, G3 e G6, como também do G5 em relação ao G1 e G6 (F(5;29)=13,88; p<0,0001). No MPE, notou-se diminuição significativa nas ratas de G4 em relação às outras (F(5;29)=10,72; p<0,0001) (Figura 1).

**Figura 1:** Espessura da cartilagem articular superior da tíbia, comparando-se as ratas distribuídas entre os grupos de estudo (G1, G2, G3, G4, G5 e G6) e os membros posteriores direito (alvo) e esquerdo (controle). Letras iguais significam semelhanças e letras diferentes significam diferenças significativas entre os grupos experimentais, para o mesmo lado

MPD: membro posterior direito; MPE: membro posterior esquerdo.

### Espessura da placa epifisária

Na análise da espessura da placa epifisária, observou-se diminuição significativa do MPD em relação ao MPE em G5 (p=0,0187). Na comparação intergrupos para o MPD, notou-se redução significativa do G5 em relação aos outros grupos, além de maiores valores em G1 com relação ao G4 (F(5;29)=14,72; p<0,0001) (Figura 2).

**Figura 2:** Espessura da placa epifisária da tíbia, comparando-se as ratas distribuídas entre os grupos de estudo (G1, G2, G3, G4, G5 e G6) e os membros posteriores direito (alvo) e esquerdo (controle). Letras iguais significam semelhanças e letras diferentes significam diferenças significativas entre os grupos experimentais, para o mesmo lado

MPD: membro posterior direito; MPE: membro posterior esquerdo.

### Número de condrócitos

Em relação ao número de condrócitos, somente em G5 houve diminuição significativa do MPD em comparação com o MPE (p=0,0006). Na comparação entre os grupos experimentais, observou-se diminuição significativa de G2, G4 e G5 em relação ao G6, e também de G1 e G3 com respeito ao G5, na comparação entre os membros posteriores direitos (F(5;29)=10,16; p<0,0001). Entre os esquerdos, houve valores maiores de G6 ao comparar com G1, G2, G4 e G5 (F(5;29)=5,11; p<0,0021) (Figura 3).

**Figura 3:** Números de condrócitos na cartilagem articular superior da tíbia (unidade), comparando-se as ratas distribuídas entre os grupos de estudo (G1, G2, G3, G4, G5 e G6) e os membros posteriores direito (alvo) e esquerdo (controle). Letras iguais significam semelhanças e letras diferentes significam diferenças significativas entre os grupos experimentais, para o mesmo lado

MPD: membro posterior direito; MPE: membro posterior esquerdo.

### Histomorfologia

Em G1 (grupo pseudo-ooforectomizado) e G4 (grupo ooforectomizado), não foram verificadas mudanças na estrutura da cartilagem articular e nem da placa epifisária, demonstrando aspectos morfológicos normais, mas, em G4, visualizou-se considerável diminuição na espessura e no número de condrócitos, como já evidenciada na análise histomorfométrica. Nas ratas de G2 (grupo pseudo- -ooforectomizado, imobilizado e com remobilização livre), foram observadas regiões de degeneração da cartilagem articular, perda da organização celular, floculações, diminuição da quantidade de condrócitos e algumas regiões com tecido de granulação (*pannus*). Já no G3 (grupo pseudo-ooforectomizado, imobilizado e remobilização em escada), notaram-se sinais de reparação das estruturas cartilaginosas, com presença de clones celulares e de *pannus*. Em G5 (grupo ooforectomizado, imobilizado e remobilização livre), foram evidenciadas degeneração da cartilagem articular, presença de fissuras e floculações, descontinuidade da *tidemark* e exposição de osso subcondral. Em G6 (grupo ooforectomizado, imobilizado e remobilização em escada), foram visualizados formação de *pannus*, invasão de vasos sanguíneos subcorticais na zona calcificada, aumento da quantidade de grupos isógenos e da espessura da zona calcificada (Figura 4).

**Figura 4:** Fotomicrografias da cartilagem articular superior da tíbia direita de ratas, G1 (A), G2 (B), G3 (C), G4 (D), G5 (E e F) e G6 (G e H); corte frontal; coloração em hematoxilina e eosina. (A) Vista panorâmica da cartilagem articular (CA), com evidência para a *tidemark* (TM) e visualização de parte de osso subcondral (OS); (B) presença de fissuras (F), floculações (Fl) e desorganização tecidual; (C) aumento da espessura da cartilagem articular, hipercelularidade e aumento do número de grupos isógenos (GI); (D) diminuição da espessura da cartilagem articular e organização celular normal; (E) perda da cartilagem articular (♦) com exposição de osso subcondral; (F) presença de floculações na superfície da cartilagem (FI); (G) recuperação da cartilagem articular (♦) com formação de *pannus* e presença de clones; (H) aumento da espessura da cartilagem articular, hipercelularidade e aumento de grupos isógenos

CA: cartilagem articular; TM: *tidemark;* OS: osso subcondral; F: fissuras; Fl: floculações; GI: grupos isógenos.

## DISCUSSÃO

Os resultados do estudo apontam que a privação hormonal estrogênica, provocada pela ooforectomia, ocasionou perda significativa de cartilagem articular. Esse hipoestrogenismo altera o processo de remodelação do tecido cartilaginoso,^(4)^ favorecendo o aparecimento da osteoartrose.^(1,5,6,14,15)^ Tal fato pode ser agravado quando há necessidade do uso de imobilização, podendo provocar lesões irreversíveis à cartilagem articular, ou seja, a associação entre a ooforectomia e imobilização pode levar mais rapidamente à instalação de prejuízos nos tecidos muscular e cartilaginoso.^(16-18)^

A imobilização provoca degeneração da cartilagem articular, com alterações atróficas, redução de sua espessura e da síntese de proteoglicanos da matriz da cartilagem, irregularidade da superfície articular, necrose e ulceração da cartilagem, aumento do número de células inflamatórias, redução da massa e volumes totais da cartilagem.^(7-9,16,19-24)^ Algumas destas características foram observadas no grupo pseudo-ooforectomizado e imobilizado, indicando efeitos agressivos da imobilização em apenas 2 semanas. Ainda, a deficiência de estrogênio leva a alterações na morfologia da cartilagem, direcionando à degeneração da mesma,^(25)^ conforme foi observado que apenas o grupo ooforectomizado apresentou valores menores, com relação à espessura da cartilagem, do membro esquerdo entre os grupos.

O exercício físico é indicado como forma de tratamento para a artralgia advinda da osteoartrite pós-menopáusica.^(26)^ Esse recurso tem sido o mais preconizado para remobilização, pois, por meio do movimento articular, é possível promover mudanças físicas, bioquímicas e histológicas que favorecem o retorno à síntese de macromoléculas e proporcionam reversibilidade, pelo menos parcial, das lesões causadas na cartilagem.^(8,27,28)^ No entanto, Portinho et al.,^(29)^ utilizando remobilização livre por 15 dias, associado a três séries, de 30 segundos, diárias, de alongamento do músculo sóleo não observaram mudanças na espessura da cartilagem articular pela imobilização e remobilização. Já Del Carlo et al.^(9)^ verificaram que a imobilização por 45 dias provocou aumento da espessura articular e do número de condrócitos, irregularidade da superfície articular e perda de proteoglicanos da matriz da cartilagem.

Não foram encontrados estudos que verificassem os efeitos sobre a cartilagem, em ratas ooforectomizadas e submetidas à imobilização, com posterior remobilização em treino de subida em escada. Porém alguns apontam sucesso desse tipo de exercício na promoção de melhora na densidade mineral óssea, na rigidez óssea e na hipertrofia dos músculos gastrocnêmio, flexor longo dos dedos, plantar e tríceps braquial.^(30-33)^

No presente estudo, foi verificado que, em ratas pseudo-ooforectomizadas e submetidas à imobilização − tanto a remobilização livre (G2) como em escada (G3) − foram capazes de manter a espessura da cartilagem articular e placa epifisária, como também o número de condrócitos, em níveis semelhantes aos do membro não imobilizado. Isso ocorreu também com as ratas ooforectomizadas, imobilizadas e em remobilização em escada (G6). No entanto, tal benefício não foi alcançado com as ratas de G5, demonstrando que a imobilização provoca perdas significativas no tecido cartilaginoso em ratas ooforectomizadas, e a remobilização livre não foi capaz de reverter os efeitos deletérios no tecido cartilaginoso, ao contrário da remobilização em escada (G6), provavelmente por requerer uma carga intensa sobre a articulação, visto que a remobilização, com descarga de peso sobre a articulação, promove a secreção de proteoglicanos na matriz celular, viabilizando a restauração da estrutura da cartilagem.^(8)^ Já foi demonstrado que o exercício em esteira mostrou-se útil em ratas ooforectomizadas quanto à proteção da cartilagem articular.^(34)^

As lesões da matriz extracelular e degradação completa da cartilagem articular podem derivar de maior atividade das metaloproteinases da matriz.^(35)^ Na análise morfológica, foi verificado predomínio de áreas degradadas, principalmente em G5, que realizou ooforectomia, imobilização e remobilização livre; e área com predomínio de recuperação da cartilagem articular, em G2, G3 e G6, sendo mais evidente nas duas últimas, apontando, assim, efeito reparador do exercício físico. Havia principalmente presença de *pannus*, aumento de grupos isogênicos e clones celulares, que decorrem da presença de inflamação dos tecidos articulares.^(36)^ Essas reações refletem um processo de recuperação frente aos danos ocasionados na cartilagem articular, visto a capacidade proliferativa dos condrócitos e reparo do osso subcondral, com aumento do aporte nutricional na região, alcançadas por meio de programas de remobilização.^(9,36)^

## CONCLUSÃO

Em suma, observou-se que a ooforectomia, associada à imobilização, provocou diminuição da espessura da cartilagem articular e placa epifisária, além de diminuição do número de condrócitos, degeneração da cartilagem articular, perda da organização celular, floculações e *pannus*. A remobilização livre não foi capaz de recuperar as lesões ocasionadas no tecido cartilaginoso da tíbia de ratas ooforectomizadas e imobilizadas, mas a reversibilidade foi alcançada com a remobilização em escada, com melhora dos parâmetros morfométricos e morfológicos, com sinais de reparação das estruturas cartilaginosas, observados pela presença de clones celulares e de *pannus*, invasão de vasos sanguíneos subcorticais na zona calcificada, aumento da quantidade de grupos isógenos e da espessura da zona calcificada.
